# Identification of prognosis-related gene features in low-grade glioma based on ssGSEA

**DOI:** 10.3389/fonc.2022.1056623

**Published:** 2022-12-14

**Authors:** Yuanzhi He, Zhangping Lin, Sanyang Tan

**Affiliations:** ^1^ Department of Neurosurgery, Wuhan Children’s Hospital (Wuhan Maternal and Child Healthcare Hospital), Tongji Medical College, Huazhong University of Science & Technology, Wuhan, Hubei, China; ^2^ Clinical Laboratory, Hainan Women and Children’s Medical Center, Haikou, Hainan, China; ^3^ Clinical Laboratory, Haikou Hospital of, The Maternal and Child Health, Haikou, Hainan, China

**Keywords:** low-grade glioma, immune infiltration, T-cell risk score, crucial gene, prognosis

## Abstract

Low-grade gliomas (LGG) are commonly seen in clinical practice, and the prognosis is often poor. Therefore, the determination of immune-related risk scores and immune-related targets for predicting prognoses in patients with LGG is crucial. A single-sample gene set enrichment analysis (ssGSEA) was performed on 22 immune gene sets to calculate immune-based prognostic scores. The prognostic value of the 22 immune cells for predicting overall survival (OS) was assessed using the least absolute shrinkage and selection operator (LASSO) and univariate and multivariate Cox analyses. Subsequently, we constructed a validated effector T-cell risk score (TCRS) to identify the immune subtypes and inflammatory immune features of LGG patients. We divided an LGG patient into a high-risk–score group and a low-risk–score group based on the optimal cutoff value. Kaplan–Meier survival curve showed that patients in the low-risk–score group had higher OS. We then identified the differentially expressed genes (DEGs) between the high-risk–score group and low-risk-score group and obtained 799 upregulated genes and 348 downregulated genes. The analysis of the Kyoto Encyclopedia of Genes and Genomes (KEGG) and Gene Ontology (GO) show that DEGs were mainly concentrated in immune-related processes. In order to further explore the immune-related genes related to prognosis, we constructed a protein–protein interaction (PPI) network using Cytoscape and then identified the 50 most crucial genes. Subsequently, nine DEGs were found to be significantly associated with OS based on univariate and multivariate Cox analyses. It was further confirmed that CD2, SPN, IL18, PTPRC, GZMA, and TLR7 were independent prognostic factors for LGG through batch survival analysis and a nomogram prediction model. In addition, we used an RT-qPCR assay to validate the bioinformatics results. The results showed that CD2, SPN, IL18, PTPRC, GZMA, and TLR7 were highly expressed in LGG. Our study can provide a reference value for the prediction of prognosis in LGG patients and may help in the clinical development of effective therapeutic agents.

## Introduction

A glioma is a tumor arising from the carcinogenesis of glial cells in the brain and spinal cord and is characterized by a high incidence, a low cure rate, aggressive growth, a high malignancy, and a significantly higher incidence in men than in women. Gliomas are commonly found in clinical practice, accounting for approximately 81% of intracranial malignancies (1). Most types of gliomas have a poor prognosis due to their malignant biological behavior (2). Low-grade gliomas (LGG) are gliomas of low malignancy, and even though the prognosis is better than that of glioblastoma, many LGG progress to high-grade gliomas due to the great heterogeneity between different LGG, and thus the prognosis of LGG patients is poor (3, 4). Currently, clinical treatment for LGG includes surgical resection, radiotherapy, chemotherapy, electric field therapy, and supportive therapy, all of which help to prolong survival time and improve quality of life (5). However, reliable biomarkers that can predict poor prognoses in patients with LGG are uncommon in the diagnosis and treatment of LGG. Therefore, a search for effective prognostic predictors and therapeutic targets for LGG is necessary.

The development of LGG is closely related to immunity, and LGG cells can secrete a large number of cytokines that promote the entry of various immune cells into the tumor, which in turn creates a tumor microenvironment (6). The tumor microenvironment is involved in LGG growth, recurrence, invasion, and response to therapy, and immune cells are closely associated with poor prognoses in LGG patients (7). Currently, immuno-oncology is of great clinical interest due to its specific benefits in the treatment of a variety of cancers. Immune-related genes and immune-infiltrating cells play an integral role in the tumor microenvironment, helping to determine prognosis and providing an impetus for immunotherapy (8). Therefore, it is crucial to find immune-related risk scores and immune-related targets for predicting prognosis.

Currently, there is no report on the bioinformatics analysis of immune-related targets for predicting LGG prognosis based on single-sample gene set enrichment analysis (ssGSEA) and constructing T-cell risk score (TCRS), as described in the previous articles. In the present study, immune cell abundance in LGG samples was explored using ssGSEA to construct an effector TCRS. The prognostic value of immune cells for overall survival (OS) time prediction was assessed to elucidate functional differences between high- and low-risk groups distinguished by TCRS. Subsequently, a protein–protein interaction (PPI) network, univariate and multivariate Cox analyses, batch survival analysis, and nomogram prediction model can identify the crucial genes related to LGG prognosis and resolve the mode of action of crucial genes in LGG, providing a reference for improving the prognosis of LGG patients.

## Data

### Data sources

RNA-seq data from the LGG patient sample (*n* = 512) and corresponding clinical information can be obtained from The Cancer Genome Atlas (TCGA) for inclusion in subsequent analyses.

### Prognostic value of immune cells for predicting overall survival

The ssGSEA algorithm is based on a set of 22 immune genes, including genes associated with different immune cell types, functions, pathways, and checkpoints. The ssGSEA results were analyzed using the R package “GSVA” to identify different levels of infiltration of immune cell types, immune-related functions, and immune-related pathways in LGG expression profiles. The image clustering heat map was drawn using the R package “Pheatmap.”

The prognostic value of 22 immune cells for predicting OS was assessed using least absolute shrinkage and selection operator (LASSO) and univariate and multivariate Cox analyses. The TCRS was determined based on the most prognostically significant immune cells, and the optimal cutoff value of the TCRS was calculated using the R package “ggrisk.” The LGG samples were divided into a high-risk–score group and a low-risk–score group based on the optimal cutoff value. Kaplan–Meier and ROC analyses were used to compare the survival of patients in both groups. Based on the ESTIMATE algorithm, the R package “ESTIMATE” was used to calculate the stroma score, immune score, and ESTIMATE score of LGG samples in different risk score groups. The box plot was drawn using the R package “ggpubr.” The relationship between different risk scores and changes in the expression of each immune gene was analyzed.

### Differential analysis

Differential analysis was performed on the high-risk–score group and a low-risk–score group using the R package “limma” according to the screening threshold of |Log2FC|>1, adj.*p* < 0.05. The R package “clusterProfiler” was used to select datasets from the Kyoto Encyclopedia of Genes and Genomes (KEGG) and Gene Ontology (GO).

### Protein–protein interaction networks

PPI networks were identified using the STRING database, and the biomolecular interaction networks of related genes were visualized using Cytoscape. A modular analysis of the network was performed using the MCODE plugin to screen the crucial genes in the PPI network. The prognostic value of the differentially expressed genes (DEGs) was analyzed using univariate and multivariate Cox analyses to obtain the genes related to the prognosis of the LGG patient sample, and *p* < 0.05 was considered to be significant.

### Identification of low-grade glioma prognosis-associated genes

A univariate Cox regression analysis was performed on the LGG patient sample DEGs to screen for genes associated with prognosis using the log-rank test. The prognostic differences between the high gene expression group and low gene expression group were analyzed using the R package “Kaplan–Meier,” and *p* < 0.05 was considered to be significant.

### Nomogram construction

Independent prognostic factors were determined based on univariate and multivariate Cox regression models. Column plots were constructed using the R packages “rms” and “survival.” A calibration curve was used to assess the accuracy of the line graphs, and *p* < 0.05 was considered to be significant.

### Cell culture

Human glioma cell lines HS683 and the normal human glial cell line HEB were selected and provided by Xiangya Medical College of Changsha Central South University (China). The cells were cultured in DMEM supplemented with 1% streptomycin/penicillin and 10% FBS under saturated humidity, 37°C, and 5% CO_2_.

### Real-time quantitative polymerase chain reaction

RNA was extracted using a TRIzol kit (Invitrogen, USA), and cDNA was synthesized using a reverse transcription kit. Real-time quantitative polymerase chain reaction (RT-qPCR) was performed using a SYBR Premix Ex Taq kit (TaKaRa, Japan). The primer sequences used are listed in **Table 1**. Gene expression levels were quantified using the 2^−ΔΔCT^ method. The experiment was repeated three times.

**Table 1:** RT-qPCR primer sequence.

Gene	Forward 5′→3′	Reverse 5′→3′
CD2	TCAAGAGAGGGTCTCAAAACCA	CCATTCATTACCTCACAGGTCAG
SPN	GCTGGTGGTAAGCCCAGAC	GGCTCGCTAGTAGAGACCAAA
IL18	TCTTCATTGACCAAGGAAATCGG	TCCGGGGTGCATTATCTCTAC
PTPRC	ACCACAAGTTTACTAACGCAAGT	TTTGAGGGGGATTCCAGGTAAT
GZMA	TCTCTCTCAGTTGTCGTTTCTCT	GCAGTCAACACCCAGTCTTTTG
TLR7	TCCTTGGGGCTAGATGGTTTC	TCCACGATCACATGGTTCTTTG
GAPDH	CTGGGCTACACTGAGCACC	AAGTGGTCGTTGAGGGCAATG

## Results

### Prognostic value of immune cells for predicting overall survival

**Figure 1:**
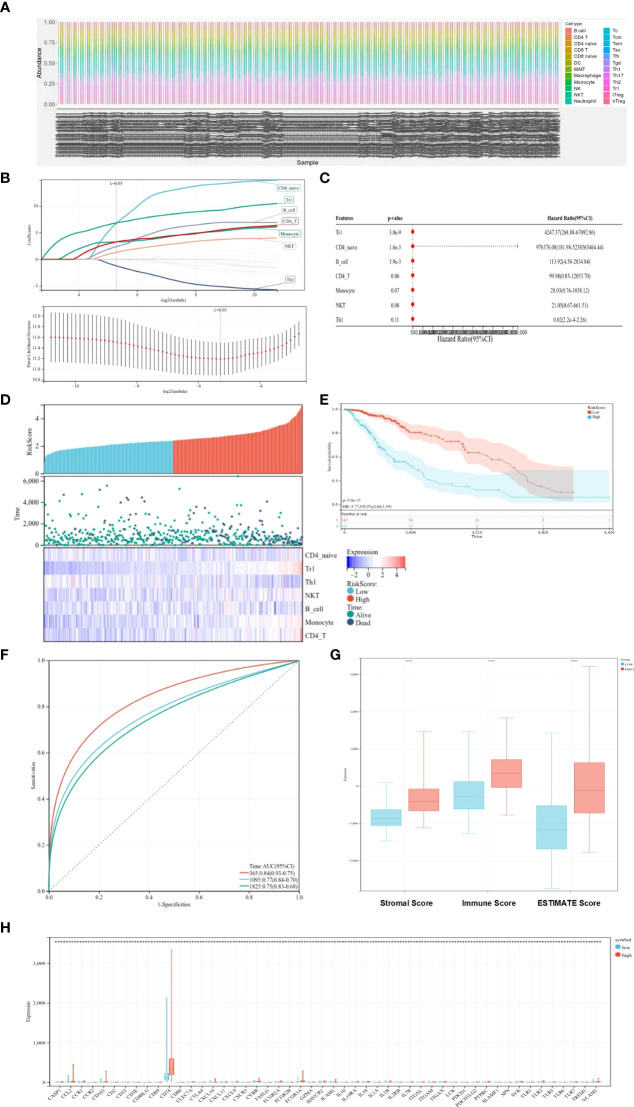
Prognostic value of immune cells for predicting OS. **(A)** Distribution of immune cell types. **(B)** LASSO coefficient profiles. **(C)** Univariate and multifactorial Cox analyses used to identify OS-related immune cells. **(D)** Heat map of survival status and expression in different risk score groups. **(E)** Kaplan–Meier survival curves showing survival in the different risk score groups. **(F)** ROC curve showing the predictive value of TCRS for 1-, 3-, and 5-year OS. **(G)** Box plots showing differences in the stromal score, immune score, and ESTIMATE score between different risk score groups. **(H)** Expression of immune genes in different risk-score groups compared with low group. ^*^ *p* < 0.05; ^**^ *p* < 0.01; ^***^ *p* < 0.001; ^****^ *p* < 0.0001.

The ssGSEA method was applied to the transcriptome of LGG samples to find the distribution of 22 immune cell types (**Figure 1A**). Based on the LASSO model, the TCRS can be calculated as follows when lambda.min = 0.0257: RiskScore = 6.74934179805055 * CD4_naive + 6.83207575533917 * Tr1 − 1.22576340775653 * Th1 + 1.31766352136828 * NKT + 2.38814433064296 * B_cell + 3.26414059327394 * Monocyte + 3.2630292767646 * CD4_T (**Figure 1B**). Furthermore, using univariate and multivariate Cox analyses, the optimal cutoff value of 2.649 was obtained, and the results showed that Tr1, CD4_naive, and B_cell were related to the OS of patients with LGG (**Figure 1C**). The LGG patient sample was divided into a high-risk–score group and a low-risk–score group based on the optimal cutoff value (**Figure 1D**). Kaplan–Meier survival curves figured out that patients in the low-risk–score group had greater OS than those in the high-risk–score group (**Figure 1E**, *p* < 0.05). The ROC curves showed that TCRS had a better predictive value for 1-, 3-, and 5-year OS (**Figure 1F**). To verify the validity of the above model, the ESTIMATE algorithm was used to calculate the stromal score, immune score, and ESTIMATE score of LGG samples. The results showed that the stromal score, immune score, and ESTIMATE score were higher in the high-risk–score group than in the low-risk–score group (**Figure 1G**, *p* < 0.05). There was a significant difference in the expression of immune genes between the high-risk–score group and the low-risk–score group (**Figure 1H**, *p* < 0.05).

### Analysis of differences

**Figure 2:**
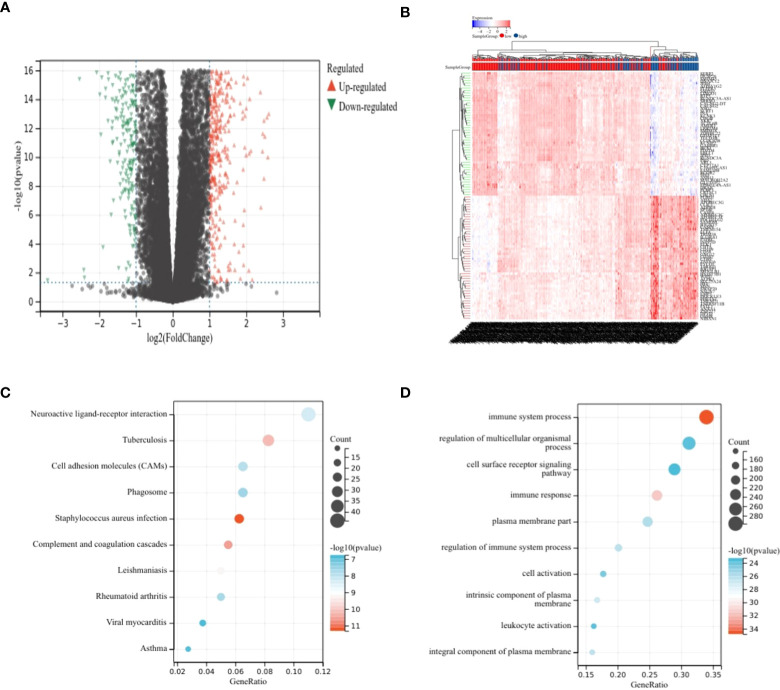
Analysis of differences. **(A, B)** Volcano plot showing DEGs in different risk score groups. **(C)** Bubble plot from KEGG analysis showing major enrichment of DEGs. **(D)** Bubble plot from GO analysis showing major enrichment of DEGs.

Differential analysis was performed on the high-risk–score group and the low-risk–score group, and 799 upregulated genes and 348 downregulated genes were identified (**Figures 2A, B**). KEGG analysis showed that DEGs were enriched mainly in *Staphylococcus aureus* infection, complement and coagulation cascades, tuberculosis, and other pathways (**Figure 2C**). GO analysis showed that DEGs were enriched mainly in the immune system process, immune response, and other terms (**Figure 2D**).

### Establishment of protein–protein interaction network

**Figure 3:**
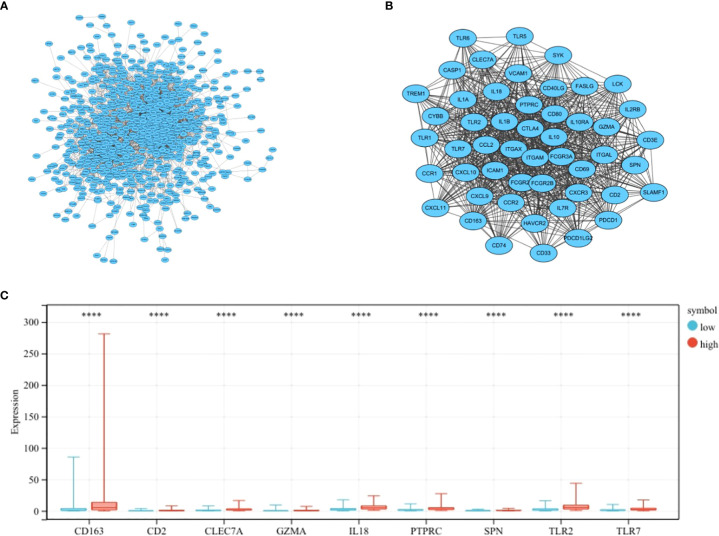
Establishment of the PPI network. **(A)** PPI network showing protein interactions between nodes. **(B)** Most important modules in the PPI network. **(C)** Expression of nine prognosis-related genes in the different risk-score groups compared to low group. ^****^ *p* < 0.0001.

The most important module in the PPI network consisted of 50 nodes and 868 edges (**Figures 3A, B** **)**. The prognostic value of the 50 DEGs was analyzed using univariate and multivariate Cox analyses, and a total of nine prognostic-related genes were obtained, including CD2, SPN, IL18, CLEC7A, PTPRC, TLR2, GZMA, CD163, and TLR7 (**Tables 2**, **3**). The expression of all nine genes was different in the different risk-score groups (**Figure 3C**, *p* < 0.05).

**Table 2:** Univariate Cox regression analysis.

Tag	HR	Lower 95%	Upper 95%	Likelihood	Log-rank	Wald
PDCD1LG2	1.520116292	1.369792153	1.686937347	7.34E−10	8.80E−17	3.20E−15
CD2	1.452943277	1.311213594	1.609992586	2.66E−08	1.18E−15	9.75E−13
CD40LG	75.90563174	23.15108433	248.8723573	2.02E−08	3.88E−15	8.93E−13
CASP1	1.213978947	1.154007576	1.277066905	4.51E−10	7.95E−15	6.31E−14
LCK	3.6385036	2.5422305	5.207516961	2.25E−08	9.35E−15	1.66E−12
SPN	2.622728033	2.023027009	3.400202915	9.09E−11	5.14E−14	3.36E−13
CD3E	1.662195837	1.43668872	1.923099249	9.79E−08	1.11E−13	8.44E−12
CXCR3	7.589077041	4.178056523	13.78489975	6.80E−08	3.83E−13	2.83E−11
CXCL11	1.181252395	1.120596448	1.245191542	2.70E−06	1.01E−12	5.89E−10
FCGR2B	1.976307546	1.61004357	2.425891813	1.48E−06	2.41E−12	7.31E−11
CCR2	4.555315166	2.836910836	7.314609962	3.53E−07	2.11E−11	3.49E−10
SLAMF1	84.49490994	19.95938776	357.6958317	1.67E−06	7.08E−11	1.68E−09
FASLG	132.1604317	24.87050552	702.29291	5.23E−06	9.26E−10	9.99E−09
IL7R	1.275505655	1.157951312	1.404994027	0.000466979	2.21E−09	8.11E−07
IL18	1.142776013	1.092357372	1.195521767	1.01E−07	2.84E−09	6.75E−09
FCGR2A	1.084849239	1.055299647	1.115226254	8.86E−07	4.07E−09	7.47E−09
CLEC7A	1.216203839	1.13925352	1.29835173	2.67E−06	4.27E−09	4.37E−09
CD80	4.563367614	2.473199935	8.419992129	0.000541267	7.15E−09	1.19E−06
IL10	1.972319731	1.548931374	2.511438005	4.07E−05	1.49E−08	3.61E−08
ITGAX	1.116224672	1.073805236	1.160319839	7.99E−07	1.49E−08	2.66E−08
PDCD1	2.892951433	1.970626433	4.24695815	1.00E−05	2.01E−08	5.86E−08
CXCL10	1.027817602	1.016827153	1.038926841	9.43E−05	3.30E−08	5.67E−07
CD69	1.39826594	1.230533292	1.588862041	1.36E−05	1.79E−07	2.72E−07
PTPRC	1.111688395	1.066629168	1.158651128	2.33E−05	3.24E−07	5.29E−07
ITGAL	1.210034564	1.121282744	1.305811272	2.64E−05	5.14E−07	9.33E−07
CD74	1.000982762	1.000574112	1.001391579	4.37E−05	1.55E−06	2.42E−06
HAVCR2	1.060795489	1.035001734	1.087232063	1.48E−05	1.93E−06	2.61E−06
TLR2	1.061560111	1.034981622	1.088821139	0.000124234	3.77E−06	3.88E−06
CXCL9	1.085836678	1.042788787	1.130661652	0.00175701	1.48E−05	6.61E−05
TLR1	1.22612022	1.11399963	1.349525397	0.0001822	2.26E−05	3.09E−05
TLR6	1.706751561	1.32659079	2.195854905	0.000152853	2.84E−05	3.21E−05
GZMA	1.207293281	1.097999303	1.327466295	0.001108677	4.71E−05	9.98E−05
IL2RB	1.601590896	1.2462979	2.058170359	0.001969111	0.000133686	0.000232795
CTLA4	2.287753151	1.470110919	3.560149383	0.004282951	0.0001396	0.000244672
CCR1	1.057674123	1.027356743	1.08888617	0.001114016	0.000179795	0.000157572
CD163	1.008029664	1.003566842	1.012512332	0.004829108	0.000181982	0.00041132
VCAM1	1.018221134	1.007751055	1.028799993	0.002950449	0.000549683	0.000616844
ITGAM	1.124513823	1.051511646	1.202584244	0.001181054	0.000597246	0.000611085
IL10RA	1.076085559	1.031286061	1.122831167	0.003457336	0.000630622	0.000725157
CD33	1.585029901	1.213991796	2.069470154	0.001298347	0.000677602	0.000711565
IL1A	1.439369434	1.15739573	1.790039752	0.005193204	0.000983137	0.001060541
CCL2	1.008871682	1.003453323	1.014319299	0.005649978	0.00101128	0.001306026
FCGR3A	1.006650517	1.002547706	1.010770119	0.005185133	0.001310516	0.001467292
CYBB	1.01873458	1.006276509	1.031346887	0.006484694	0.003259481	0.003110249
ICAM1	1.025125663	1.006887071	1.043694626	0.019545403	0.005367287	0.006742245
TLR7	1.099084271	1.024661061	1.178912989	0.013378943	0.008035934	0.008267125
SYK	1.060559802	1.015264986	1.10787539	0.011451597	0.008064287	0.008284398
TLR5	1.185162561	1.033717809	1.358794715	0.024620574	0.015399611	0.014876759
TREM1	1.142466409	1.003308082	1.300925927	0.087955159	0.04046097	0.044452378

**Table 3:** Multivariate Cox regression analysis.

Tag	Exp(coef)	*p*-value	Lower 95%	Upper 95%	Coef
CD2	4.411465527	0.036529593	1.0974606	17.73278066	1.484206953
SPN	5.049279253	0.001727158	1.833888852	13.90227164	1.619245511
IL18	1.253022008	0.005611449	1.068163609	1.469872349	0.22555824
CLEC7A	0.672503094	0.042080937	0.458728497	0.985899969	−0.396748567
PTPRC	1.476656053	0.003460154	1.137091104	1.917623919	0.389780108
TLR2	0.839361265	0.021333758	0.723100033	0.974315172	−0.175114075
GZMA	0.147221226	0.000464471	0.050361944	0.430366421	−1.915818884
CD163	1.029691184	0.010471213	1.006880376	1.05301877	0.029258936
TLR7	0.656053832	0.039488752	0.439226813	0.979918843	−0.421512432

### Batch survival analysis to identify low-grade glioma prognosis-related genes

**Figure 4:**
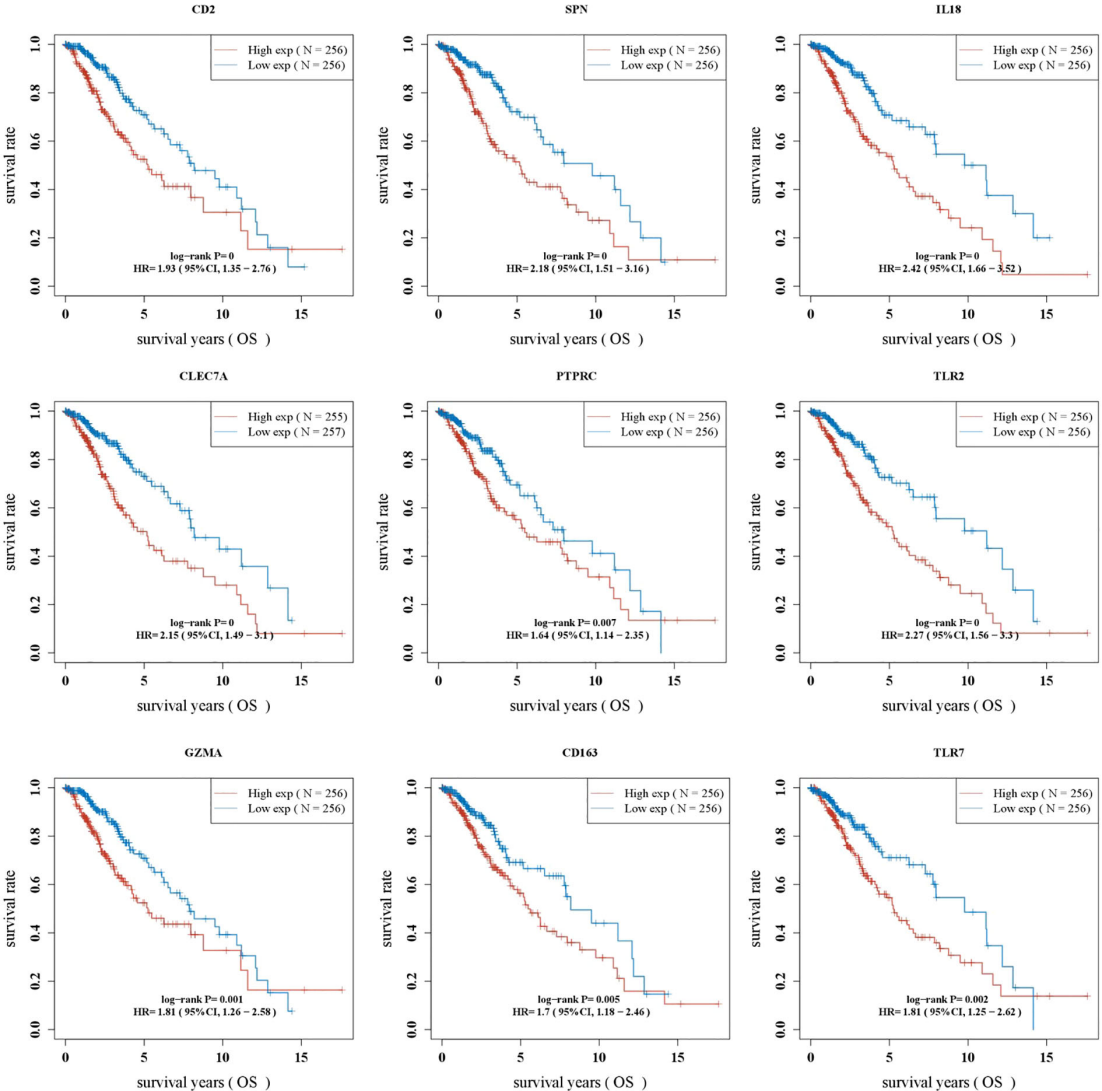
Batch survival analysis identifying LGG prognosis-related genes.

Kaplan–Meier survival curves showed that OS was significantly worse in the group with a high expression of CD2, SPN, IL18, CLEC7A, PTPRC, TLR2, GZMA, CD163, and TLR7 (**Figure 4**, *p* < 0.05).

### Construction of nomogram prognostic model

**Figure 5:**
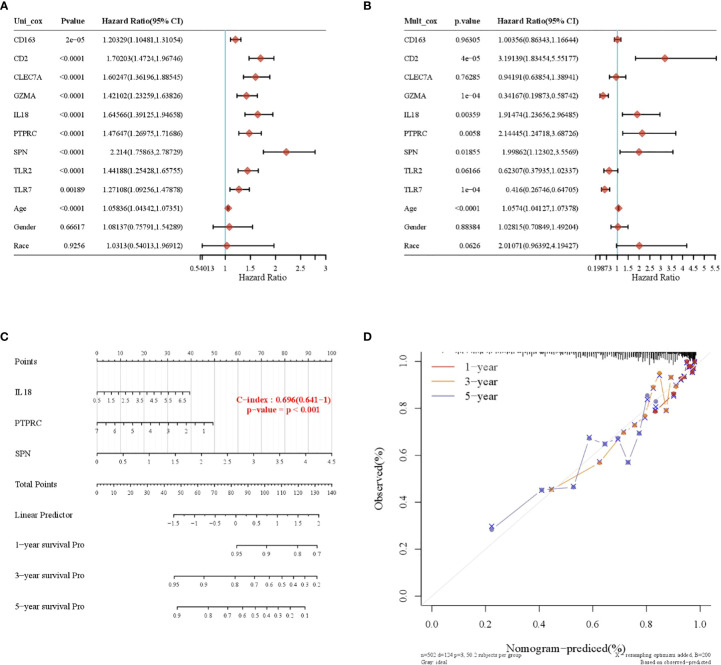
Construction of a nomogram prognostic model. **(A)** Univariate Cox regression model. **(B)** Multivariate Cox regression model. **(C)** Column-line plot for predicting survival time. **(D)** Calibration curve for predicting a 1-, 3-, and 5-year prognosis.

A total of nine prognosis-related genes, age, sex, and race were used as variables, and univariate and multivariate Cox analyses showed that CD2, SPN, IL18, PTPRC, GZMA, and TLR7 were independent prognostic factors for LGG (**Figures 5A, B**, *p* < 0.05). The nomogram prediction model had good predictive power for 1-, 3-, and 5-year prognoses in LGG patients (**Figures 5C, D**).

### Expression validation of crucial genes

**Figure 6:**
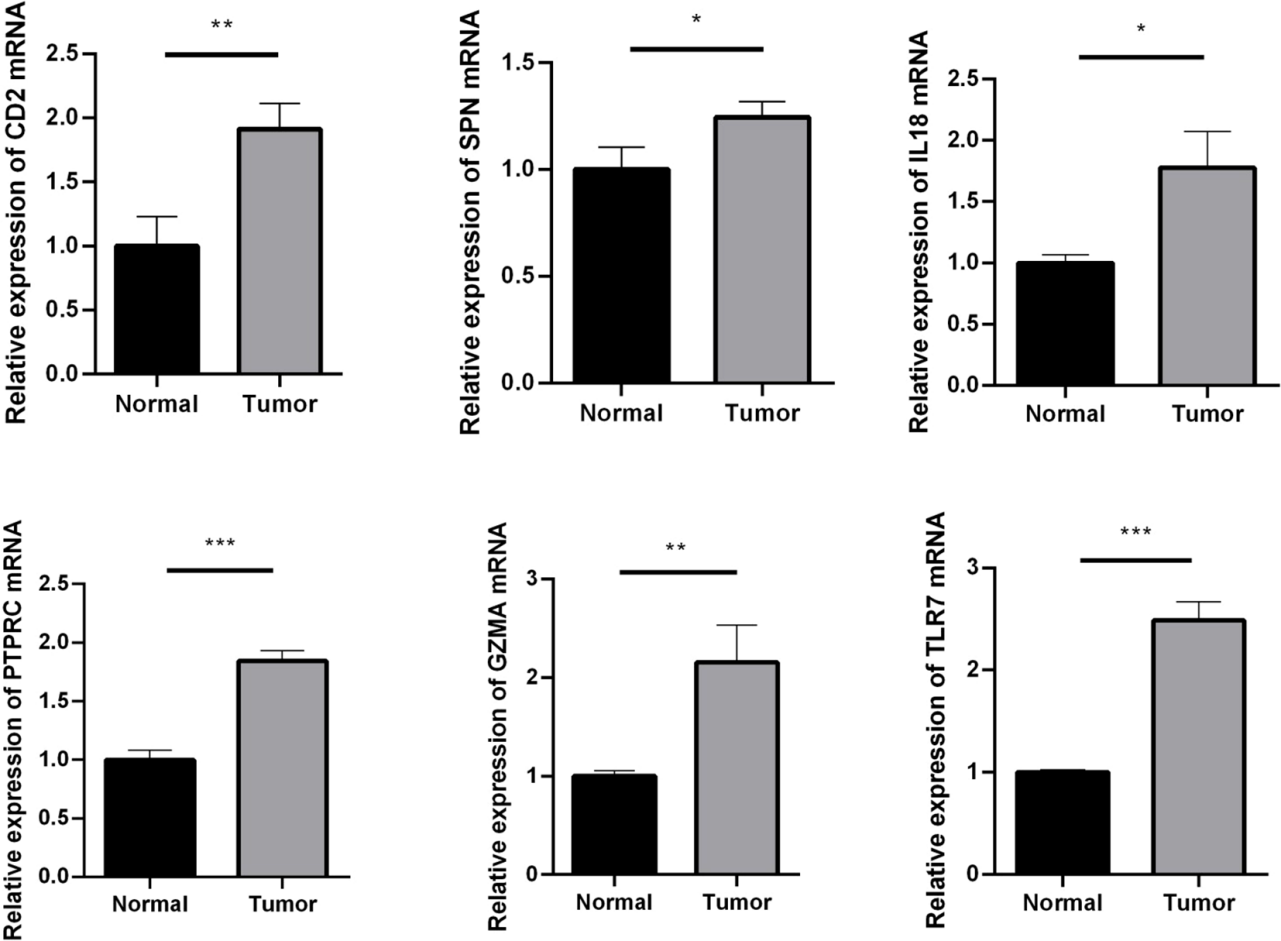
RT-qPCR assay used to verify the expression of crucial genes. Compared with the normal group. ^*^ *p* < 0.05; ^**^ *p* < 0.01; ^***^ *p* < 0.001.

CD2, SPN, IL18, PTPRC, GZMA, and TLR7 genes were validated using an RT-qPCR assay. The results showed that the expression of CD2, SPN, IL18, PTPRC, GZMA, and TLR7 was upregulated in the tumor group compared with the normal group (**Figure 6**, *p* < 0.05).

## Discussion

LGG have become a focus of brain tumor research. Surgical resection is currently the main treatment modality for LGG, but because LGG frequently presents with infiltrative growth and is mostly found in functional areas of the brain, extended resection by surgery is greatly limited, and the LGG patients still do not have an ideal prognosis (9, 10). As a result, there is a need to construct effective prognostic prediction models for LGG. Immune infiltration plays a role in the development of LGG (11). Because LGG survive in a complex tumor microenvironment, it brings a serious challenge for clinical assessment and treatment of LGG.

Wu et al. constructed an immune risk score signature (IRSS) using the LASSO model, and the IRSS included six relevant immune genes that were good predictors of prognosis in LGG patients. Moreover, the immune infiltration results showed that the genetic profile correlated with innate immune cytopenia (12). Zhang et al. found that using LASSO and multivariate Cox regression analyses, they were able to obtain six immune genes that comprise a risk model and may be involved in the process of neoantigen presence and triggering immune responses (13). The present study differs from previous LGG-related literature in that we obtained crucial genes that may be associated with LGG prognosis based on ssGSEA and by constructing TCRS. In this study, ssGSEA was performed on 22 immune gene sets to calculate immune-based prognostic scores. The prognostic value of the 22 immune cells for predicting OS was assessed using LASSO and univariate and multivariate Cox analyses. Subsequently, we constructed a validated TCRS to identify immune subtypes and inflammatory immune features in LGG patients. Trl, CD4_naive, and B_cells were found to be related to OS in LGG patients by LASSO and by univariate and multifactorial Cox analyses. We divided the LGG patient sample into a high-risk–score and low-risk–score group according to the optimal cutoff value. Kaplan–Meier survival curves displayed that patients in the low-risk–score group had higher OS. The ROC curve showed that TCRS was able to identify the immune subtype of LGG and had a better predictive value for 1-, 3-, and 5-year OS. We then identified DEGs in the high-risk–score and low-risk–score groups and obtained 799 upregulated genes and 348 downregulated genes. KEGG and GO analyses showed that DEGs were enriched mainly in immune-related processes. We constructed a PPI network using Cytoscape and then identified the top 50 crucial genes. Subsequently, nine DEGs were found to be significantly related to OS based on univariate and multivariate Cox analyses. OS was significantly worse in the high-expression group of CD2, SPN, IL18, CLEC7A, PTPRC, TLR2, GZMA, CD163, and TLR7 compared to the low-expression group. This indicates that these nine crucial genes may be related to the process of immune cells affecting OS. Finally, we constructed a prognostic nomogram model that revealed CD2, SPN, IL18, PTPRC, GZMA, and TLR7 to be independent prognostic factors for LGG. Columnar plots and ROC curves were used to verify that the model was reasonably accurate in predicting the prognosis of LGG patients at 1, 3, and 5 years. In addition, this study used an RT-qPCR assay to verify the bioinformatics results, revealing that CD2, SPN, IL18, PTPRC, GZMA, and TLR7 were highly expressed in LGG.

CD2 is expressed on the surface of all peripheral blood T cells, more than 95% of human thymocytes, most NK cells, and some malignant B cells and may indirectly reflect the immune function of the body’s cells (14). Chen’s team found that CD2 was upregulated in breast cancer samples and that CD2 immunomodulation contributed to the mitigation of disease progression and could be used as an immunomodulatory agent in clinical treatment (15). SPN, alias CD43, encodes a glycoprotein that is expressed on the membrane surface of normal and tumorigenic T cells. SPN can regulate intercellular adhesion, intracellular signaling, cell proliferation, and apoptosis (16). Gao’s team found that miR-129-5p was beneficial in delaying the malignant progression of clear cell renal carcinoma by targeting the downregulation of SPN (17). IL18 is a proinflammatory cytokine with important functions, such as induction of angiogenesis and regulation of immune function, and is involved in the progress of many inflammatory diseases, immune disorders, and tumors (18). Park’s team speculated that IL18 contributes to the poor prognosis of triple-negative breast cancer patients by inducing immunosuppression of PD-1 expression on NK cells (19). PTPRC, alias CD45, is an antigen of leukocytes that is common on their surface. PTPRC acts as a key molecule of signal transduction on cell membranes and positively regulates T-cell antigen receptor signaling (20, 21). PTPRC can affect the processes of cell growth, differentiation, and mitosis, and it has been suggested that PTPRC may have a role in regulating the MAPK/ERK signaling pathway, with implications for cervical carcinogenesis and patient prognosis (22). GZMA is a serine protease that is mainly secreted by NK cells and cytotoxic T lymphocytes and delivered to bacterial or virally infected target cells (23). GZMA mediates apoptosis and cell scorching, induces the release of inflammatory factors, is involved in the body’s defense against pathogenic bacterial infections, and is associated with the development of certain autoimmune diseases (24). Santiago’s team showed that GZMA can be involved in tumor development and is a potential prognostic target for various cancers; this may be due to the ability of extracellular GZMA to promote the production of NF-κB-dependent IL6 in macrophages (25). TLR7 is an important pattern recognition receptor in natural immunity, playing a vital role in the body’s resistance to pathogenic infections and acting as a key line of defense for the immune system (26). Studies have shown that TLR7 can be used as a reliable marker of poor prognosis, which reveals the high expression of TLR7 in patients with non-small cell lung cancer, which is related to the inflammatory process of TLR7 signaling (27).

In summary, CD2, SPN, IL18, PTPRC, GZMA, and TLR7 were identified as independent prognostic factors for LGG, and these genes may be potential indicators of the regulation of the immune microenvironment. This may contribute to the clinical development of more effective therapeutic agents. To ensure the accuracy of the results, a larger sample is needed, and other datasets should be used for validation. In addition, the mechanisms of action of CD2, SPN, IL18, PTPRC, GZMA, and TLR7 in LGG require further exploration.

## Data availability statement

Publicly available datasets were analyzed in this study. These data can be found in the TCGA database (https://tcga-data.nci.nih.gov/tcga/).

## Author contributions

YH: Conceptualization, Methodology, Formal analysis, Writing - Original Draft. ZL: Methodology, Formal analysis, Writing - Review & Editing. ST: Conceptualization, Validation, Data Curation, Writing - Original Draft. All authors contributed to the article and approved the submitted version.
